# Simultaneous Bilateral Total Knee Arthroplasty in Elderly: Are There Factors that Can Influence Safety and Clinical Outcome?

**DOI:** 10.1155/2022/1989822

**Published:** 2022-08-21

**Authors:** G. Piovan, D. Screpis, S. Natali, V. Iacono, M. Baldini, L. Farinelli, M. Guerriero, C. Zorzi

**Affiliations:** ^1^IRCCS Ospedale Sacro Cuore—Don Calabria, Viale Luigi Rizzardi 4, Negrar di, Valpolicella (VR), Italy; ^2^Clinica Ortopedica Dell'adulto e Pediatrica, Università Politecnica Delle Marche, Via Tronto 10/A, Ancona (AN), Italy; ^3^Dipartimento di Statistica Applicata, Università Degli Studi di Verona, Via S. Francesco, 22, Verona (VR), Italy

## Abstract

**Objective:**

The aim of this study was to look for preoperative patients' related factors correlating with worse clinical outcomes in a cohort of elderly patients undergoing simultaneous bilateral total knee arthroplasty (SiBTKA) to search for risk factors, which may influence clinical outcomes and safety. *Subjects and Methods*. The hospital database was mined searching for patients older than 70 years that underwent SiBTKA for severe bilateral knee osteoarthritis (OA) between 2012 and 2016. Preoperative clinical information, Oxford Knee Score (OKS), and Knee Injury and Osteoarthritis Outcome Score (KOOS) prior to surgery were recorded. The OKS and the KOOS were submitted again after a minimum of 5 years of follow-up (FU).

**Results:**

An improvement was observed in all clinical scores at last FU. The major complication rate was 5.4%. No patients' clinical data showed correlation with perioperative complications or need for transfusions. Functional scores at the last FU were negatively affected by age at surgery and positively affected by preoperative clinical scores. *Discussion*. In the setting of severe symptomatic bilateral knee OA, SiBTKA seems to be effective in improving symptoms at midterm follow-up, with acceptable rates of perioperative complications in patients older than 70. Higher age at surgery and lower preoperative functional scores are associated with worse clinical outcomes at FU. This could assist surgeons in advising patients that delay of surgical treatment could worsen outcomes.

## 1. Introduction

Osteoarthritis (OA) is the most prevalent joint disease and a leading source of chronic pain and disability in the developed world, and its prevalence is growing along with life expectancy [1].

Approximately, 20% of patients affected by knee OA have severe pain in both knees and 10% of patients undergo contralateral total knee arthroplasty (TKA) within 1 year from the first procedure [2].

Simultaneous bilateral total knee arthroplasty (SiBTKA) has been proposed as an alternative in which both knees are replaced at the same time, under the same anaesthesia by two cooperating surgical teams. It is estimated that in the US, around 700.000 TKA are performed every year [3], 6% of which are bilateral (BTKAs) and are carried out simultaneously [4].

Several studies have demonstrated that SiBTKA is economically advantageous compared to staged unilateral surgery because of the shorter hospital stay and functional recovery times [5, 6], but there seems to be an increased risk of postoperative complications including cardiopulmonary complications, deep vein thrombosis (DVT), and pulmonary embolism (PE) [7]. On the other hand, it is also known that selected subpopulations of patients who are at greater risk for perioperative complications would be exposed to increased risks of recurrence with a staged surgery, and some of them would potentially lose the possibility to complete the bilateral TKA [8].

Cahill et al. found that SiBTKA can be effective and sufficiently safe also in octogenarian, reporting rate of complications comparable to that of standard UTKA [4]. In addition, a systematic review from Malahias et al. concluded that SiBTKA is as safe as StBTKA at all age. [9].

Remily et al. collected data of patients undergoing SiBTKA between 2009 and 2016 from the national US database. They found that SiBTKA was increasingly being performed in patients with a higher BMI and lower age, reporting decreased rates of complications. They concluded that further studies should be conducted to assess the relation between “*complications and outcomes in certain patient populations*” [10].

Given the actual debate, it would be of great interest to point out preoperative data that could predict worse perioperative and postoperative outcomes in SiBTKA and that may help surgeons in patients' counselling prior to surgery. This is even more important in the setting of older patients, where the number of comorbidities and potential complications are increased. Moreover, in the upcoming era of patient specific instrumentation (PSI), [11] a single bilateral procedure could be more practical and cost-saving.

The aim of this study is to present results at midterm follow-up (FU) of the cohort of patients older than 70 years who underwent SiBTKA, analyzing the safety and efficacy of the procedure. In addition, we conducted a multivariate analysis looking for correlation between several preoperative clinical information and perioperative and postoperative outcomes and complications.

## 2. Materials and Methods

After obtaining informed consent from the patients, the database of “Sacro Cuore–Don Calabria” Hospital of Negrar was mined searching for patients who underwent simultaneous bilateral total knee arthroplasty (SBTKA) between 2013 and 2016. Retrospective analysis of medical charts was conducted.

Inclusion criteria were as follows: age at surgery >/ = 70 years, SBTKA performed for bilateral end stage primary knee osteoarthritis (OA), and patients with a minimum follow-up of 5 years.

Exclusion criteria were as follows: patients undergoing TKA for secondary OA, revision TKA, and patients with missing data from the medical chart.

### 2.1. Data Collection

Preoperative clinical data, including patients' age, sex, body mass index (BMI), Oxford Knee Score (OKS) [12] and Knee Injury and Osteoarthritis Outcome Score (KOOS–I) [13], number and type of comorbidities, levels of hemoglobin, and anesthesiological risk according to the classification system of the American Society of Anesthesiologist (ASA) were collected.

Perioperative complications evaluated during hospital stay included ischemic stroke, myocardial infarction, other cardiac complications, respiratory complications, digestive complications, urinary complications, anemia, blood transfusions, and confusion. Orthopedic complications of deep knee infection, superficial knee infection, major mechanical malfunction, and minor mechanical malfunction were evaluated during the follow-up.

The OKS, the KOOS, and orthopedic complications were recorded at the last follow-up (FU), with a minimum of 5 years FU.

A multivariate analysis was performed to evaluate whether factors such as BMI, sex, age, number of comorbidities, and preoperative hemoglobin could influence the possible need for transfusions, the onset of complications, and clinical results at the final FU.

### 2.2. Surgical Technique

All procedures were performed at the same centre by the same surgical equipment and under the supervision of the senior author C. Z.

Total knee arthroplasty (TKA) was performed simultaneously with both tourniquets inflated at the same time, double surgical equipment and instrument sets.

For all procedures a medial subvastus capsular approach was performed. Bilateral cruciate retaining (CR), posterior stabilized (PS), or mixed CR/PS implant were chosen according to surgeon preoperative and intraoperative evaluation. The same prosthesis was implanted in all cases using a coronal mechanical alignment technique.

Two drainages, one intra-articular and the other subcutaneous, were routinely used and removed within 48 hours.

For the prevention of deep vein thrombosis, a multimodal approach was employed, and each patient underwent the same rehabilitation protocol.

The hemoglobin (Hb) trigger for transfusion was 8.0 g/dL in asymptomatic patients with intermediate cardiovascular risk. A higher trigger (Hb < 9.0 g/dL) was used for patients with high cardiovascular risk and for symptomatic ones.

### 2.3. Statistical Analysis

Demographic and clinical data were summarized using descriptive statistics. Continuous data are reported as the mean and standard deviation and categorical data as absolute and relative frequencies. Two paired sample medians were compared using the Wilcoxon signed-rank test, whereas two multivariate regression models were used to analyze the total KOOS scale and the Oxford scale at the final follow-up adjusted for confounding factors. The significance level of statistical tests was fixed at *p* < 0,05. Statistical analysis was performed using STATA version. 15.

## 3. Results

After selection according to our inclusion and exclusion criteria, a total of 55 patients were included in this study. Age, sex, the type of implants, and other clinical information are reported in Table 1. A CR implant was adopted as a standard whenever possible, using PS implants only when necessary.

No comorbidity was present in 17 patients (30%) at the time of surgery, while 38 (70%) presented with at least one disease.

Patients were classified as ASA I in 4 cases (7.3%), ASA II in 41 cases (74.5%), and ASA III in 10 cases (18.2%).

The mean preoperative hemoglobin was 13.4 ± 1.29 g/dL, and the mean postoperative hemoglobin was 12,0 ± 1,28 g/dL. The number and the type of comorbidities are described in Table 2.

The values of KOOS symptoms (KOOSs), pain (KOOSp), daily life functions (KOOSdlfs), sport and activity (KOOSsa), quality of life (KOOSqol), and OKS improved significantly from the baseline to the last follow-up. The scores are summarized in Table 3.

No intraoperative complications were recorded.

Acute minor postoperative complications, including fever, hypotension, and confusion, appeared in 13 patients (29,0%), while 23 patients (41,8%) needed blood transfusions for postoperative anemia.

Postoperative hemoglobin values, collected 6 hours, 1 day, and 3 days after surgery, were 12.0 ± 1.28 g/*d*L, 10.7 ± 1.21 g/*d*L, and 9.1 ± 1.28 g/*d*L, respectively.

Severe complications were presented in 3 patients (5.4%): 2 patients (3.6%) with acute renal failure (AKI) and 1 patient (1.8%) atrial fibrillation (AF). No cases of deep vein thrombosis (DVT) nor pulmonary embolism (PE) was recorded (Table 4).

The average length of stay (LOS) in the department of orthopaedics and traumatology was 9.8 ± 3.7 days (range: 5–27), with the outlier 27 days being due to acute renal failure treated with haemodialysis and resolved.

At midterm follow-up, seven patients (12.7%) reported late onset complications, including algodystrophy syndrome, traumatic periprosthetic fracture (PF), and chronic periprosthetic joint infection (PJI). We reported only one death caused by pancreatic cancer, thus not correlated with the procedure.

The multivariate analysis shows that the postoperative clinical result, namely, the KOOS and OKS are positively influenced by the preoperative value and negatively influenced by the patient's age at surgery (Tables 5 and 6).

Functional scores and all other outcomes such as need for transfusions, number of infused blood bags, and presence of perioperative complications are not correlated with the factors taken into consideration.

## 4. Discussion

To the best of our knowledge, this is the first study correlating preoperative patients' clinical information with short and midterm clinical outcomes and complications in a selected population of elderly patients undergoing SiBTKA.

According to our results, functional scores at the last FU were negatively associated with age at surgery and positively associated with preoperative knee scores.

For each point of the preoperative KOOS, the postoperative OKS and KOOS increased, respectively, 1.4 and 1.8 points. On the other hand, the KOOS and OKS decreased, respectively, 1.6 and 0.8 points for each additional year of patient's age at surgery.

No other preoperative information was found to correlate with perioperative complications and outcomes. Sex, BMI, comorbidities, preoperative Hb, and ASA did not correlate with functional scores, postoperative complications, and transfusions.

Kozai L et al. analyzed perioperative complications in patients older than 70 years undergoing SiBTKA. Even if the number of blood transfusions was higher in patients >70 years, they found that the overall complication rate was similar, concluding that older age was not to be considered an absolute contraindication to SiBTKA [14].

Agarwala S. et al found no difference in functional outcomes and postoperative complications in obese patients undergoing SiBTKA compared to patients with a BMI of <30. Thus, they concluded that SiBTKA have comparable results both in obese and nonobese patients [15]. Similarly, our results suggest that the BMI does not correlate with functional outcomes and postoperative complications.

Hernandez et al. found no correlation between the BMI, age, and the ASA score and hospital returns at 90 days after SiBTKA [16]. In addition, according to Takagawa S and coll., the ASA score and the BMI did not interfere with postoperative rehabilitation protocols in patients undergoing SiBTKA [17].

On the other hand, Yoon et al. reported a significantly higher complication rate after SiBTKA in ASA 3 and 4 compared to low risk patients (ASA 1/2) [18]. According to our results, ASA scores showed no correlation with perioperative complications, even though the low proportion of high-risk patients in our cohort could have weakened the statistical power of the analysis.

Although many authors have reported positive outcomes in different subsets of patients [4, 9, 14, 19], some studies warn of the potential higher risk of complications after SiBTKA.

A recent meta-analysis from Warren et al. reported an increased rate of complications after SiBTKA in all groups, regardless of preoperative health status. They concluded that SiBTKA might not be safe “*even in the healthiest patients*” [20].

A meta-analysis from Restrepo et al. reported the increased number of complications for SiBTKA compared to StBTKA and UTKA. Oddly, they found an inferior incidence of deep venous thrombosis (DVT) in the SiBTKA group even though this difference was not statistically significant [21]. Other authors have proposed a score of appropriateness to identify patients at lower risk for postoperative complications after SiBTKA [22].

Given that the evidence is conflicting, there is the need to better understand the correlation between the patient's preoperative clinical information and postoperative outcomes, in order to help surgeons during preoperative patients' counselling.

In conclusion, the outcomes presented in this study show that SiBTKA appears to be effective and relatively safe, even if the design of the present study does not allow us to conclude over these features. Perioperative complications were largely of minor entities with a rate of major complications comparable to those reported in other studies after TKA [23].

No preoperative patients' clinical data were associated with perioperative complications or need for transfusions.

Higher age at surgery and lower preoperative functional scores are associated with worse functional outcomes. This information could assist surgeons in counselling patients that when SiBTKA is indicated, delaying the surgical procedure could lead to worse outcomes.

This study has some limitations: the small sample size, the presence of a small number of patients with high ASA scores, and the absence of the control group, even if the latter is consistent with the purpose of the study. Strengths of the study are the selected old age of the patients, the correlation of preoperative clinical information with both perioperative complications and functional outcomes, and the relatively long FU.

## Figures and Tables

**Table 1:** Patients' data.

Age	75.05 ± 3,83 (range 70–83)

Type of implant	50 CR (90.9%)
	1 PS (1.8%)
	4 mixed PS - CR (7.2%)

Sex	Male 20 (36.3%)
	Female 35 (63.6%)

BMI	Underweight (<18) 1 patient (1.8%)
	Normal (18.5–25) 21 patients (38.1%)
	Overweight (25–30) 20 patients (36.3%)
	Obese I (30–35) 11 patients (20%)
	Obese II (>35) 2 patients (3.6%)

ASA	I 4 patients (7.3%)
	II 41 patients (74.5%)
	III 10 patients (18.2%)
	IV No patients

Preoperative hemoglobin (mg/dL)	13.38 ± 1.32 (range 10.5–16.1)

Comorbidities	0 15 patients (30.9%)
	1 12 patients (21.8%)
	2 9 patients (16.3%)
	>3 17 patients (30.9%)

BMI: body mass index; ASA: American Score of Anesthesiologist.

**Table 2:** Patients' comorbidities.

Comorbidities	Number of patients
Hypertension	30 patients (54.5%)
Hypercholesterolemia	17 patients (31%)
Diabetes	6 patients (11%)
Hypothyroidism	5 patients (9%)
Cardiovascular diseases	5 patients (9%)
Pulmonary disease	3 patients (5.4%)
CKD	2 patients (3.6%)

CKD: chronic kidney disease.

**Table 3:** Preoperative and postoperative clinical outcomes according to the KOOS and OKS.

	Preoperative values	Last follow-up	*p* value
KOOS symptoms	65.79 ± 8.25	86.03 ± 12.48	<0.0001
KOOS pain	52.1 ± 11.06	84.87 ± 19.74	<0.0001
KOOS day living	47.15 ± 10.49	79.8 ± 22.62	<0.0001
KOOS sports	17,7 ± 9.01	32,4 ± 22.97	0.0002
KOOS quality of life	41.94 ± 14.69	73.05 ± 28.63	<0.0001
OKS	26.31 ± 9.31	38.13 ± 9.31	<0.0001

KOOS: Knee Injury and Osteoarthritis Outcome Scale; OKS: Oxford Knee Score.

**Table 4:** Postoperative complications.

	No of patients (%)
Minor complications	5 fever (9%)
	4 hypotension (7.2%)
	3 confusion (5.4%)
Major complications	2 AKI (3.6%)
	1 AF (1.8%)
Long term complications	4 algodystrophy syndrome (7.2%)
	2 traumatic PF (3.6%)
	1 chronic PJI (1.8%)

AKI: acute kidney injury; AF: atrial fibrillation; PF: periprosthetic fracture; PJI: periprosthetic joint.

**Table 5:** Correlation between the preoperative data and postoperative KOOS. The preoperative KOOS was positively associated with the postoperative KOOS, while age was negatively associated.

	Coef.	*p *>* *|t|	(95% C I)
Preoperative KOOS	1.83	<0.001	1.32; 2.34
Sex	2.27	0.658	−8.24; 12.78
Age	−1.63	0.030	−3.08; -0.17
BMI	0.53	0.441	−0.87; 1.93
Preoperative Hb	−1.35	0.679	−8.05; 5.35
No of comorbidities	−1.12	0.454	−4.20; 1.94
ASA	−7.60	0.256	−21.15; 5.94
Presence of complications	−5.20	0.292	−15.19; 4.79

**Table 6:** Correlation between the preoperative data and postoperative OKS. The preoperative KOOS was positively associated with the postoperative KOOS, while age was negatively associated.

	Coef.	*p *>* *|t|	(95% C I)
Preoperative OKS	1.37	<0.001	1.67; 1.68
Sex	−1.61	0.426	−5.74; 2.52
Age	−0.83	0.006	−1.39; −0.27
BMI	−0.05	0.823	−0.60; 0.48
Preoperative Hb	1.94	0.144	−0.71; 4.61
No of comorbidities	0.01	0.990	−1.21; 1.22
ASA	−3.95	0.131	−9.19; 1.28
Presence of complications	−2.40	0.212	−6.29; 1.47

## Data Availability

The datasets generated and/or analyzed during the current study are not publicly available due to respect of privacy policies but are available from the corresponding author on reasonable request and after obtaining patients' consent.
